# Endosome and Lysosome Membrane Properties Functionally Link to γ-Secretase in Live/Intact Cells

**DOI:** 10.3390/s23052651

**Published:** 2023-02-28

**Authors:** Mei C. Q. Houser, Shane P. C. Mitchell, Priyanka Sinha, Brianna Lundin, Oksana Berezovska, Masato Maesako

**Affiliations:** Alzheimer’s Disease Research Unit, MassGeneral Institute for Neurodegenerative Disease, Massachusetts General Hospital/Harvard Medical School, 114, 16th Street, Charlestown, MA 02129, USA

**Keywords:** γ-secretase, endosome and lysosome, membrane properties, Aβ

## Abstract

Our unique multiplexed imaging assays employing FRET biosensors have previously detected that γ-secretase processes APP C99 primarily in late endosomes and lysosomes in live/intact neurons. Moreover we have shown that Aβ peptides are enriched in the same subcellular loci. Given that γ-secretase is integrated into the membrane bilayer and functionally links to lipid membrane properties in vitro, it is presumable that γ-secretase function correlates with endosome and lysosome membrane properties in live/intact cells. In the present study, we show using unique live-cell imaging and biochemical assays that the endo-lysosomal membrane in primary neurons is more disordered and, as a result, more permeable than in CHO cells. Interestingly, γ-secretase processivity is decreased in primary neurons, resulting in the predominant production of long Aβ42 instead of short Aβ38. In contrast, CHO cells favor Aβ38 over the Aβ42 generation. Our findings are consistent with the previous in vitro studies, demonstrating the functional interaction between lipid membrane properties and γ-secretase and provide further evidence that γ-secretase acts in late endosomes and lysosomes in live/intact cells.

## 1. Introduction

γ-Secretase is an aspartyl protease complex responsible for the proteolytic cleavage of a wide range of transmembrane proteins within the membrane lipid bilayer [1,2]. While some γ-secretase cleavage products function as signaling molecules (e.g., Notch ICD), others may be intermediates that are destined for degradation (i.e., γ-secretase may act as “the proteasome of the membrane” [3]). The amyloid precursor protein (APP) processing is well studied in the context of Alzheimer’s disease (AD) since this substrate cleavage results in the production of β-amyloid (Aβ) [4,5]. γ-Secretase processes C99 (also known as APP CTFβ) stepwise [6,7], generating various lengths of Aβ peptides ranging from 37 to 43 amino acids. Increased and decreased γ-secretase processivity results in the generation of shorter (e.g., Aβ38) and longer Aβ species (e.g., Aβ42), respectively [8,9,10]. The accumulation of Aβ, particularly Aβ42, in the brain parenchyma is one of the pathological hallmarks of AD [11]. γ-Secretase inhibitors unexpectedly caused cognitive worsening plausibly due to their impact on other substrates [12,13]. On the other hand, recent clinical trials show that antibodies against Aβ can slow cognitive decline in AD [14,15]. Moreover emerging evidence demonstrates the causal link between inefficient C99 processing and endo-lysosomal abnormalities [16,17,18,19]. Our recent development of novel genetically encoded Förster resonance energy transfer (FRET)-based biosensors has allowed the recording of γ-secretase activity in living cells [20,21]. The principle of these biosensors is the C-terminus of human APP C99 is fused to the donor and acceptor fluorescent proteins with a flexible linker, and the “sensing” domains are stabilized near the membrane by fusion to a membrane-anchoring domain. The proteolytic processing of APP C99 within the biosensor by endogenous γ-secretase results in a change in the proximity and/or orientation between the donor and acceptor, which can be recorded as decreased FRET efficiency. Importantly, multiplexed FRET analysis utilizing the near-infrared analog has enabled “visualizing” predominant γ-secretase activity in late endosomes and lysosomes of live/intact neurons [22,23].

γ-Secretase is embedded in the membrane lipid bilayer. Therefore lipid conditions in the membrane can affect this protease’s function. Indeed, previous studies employing the proteoliposomes in which γ-secretase is reconstituted and/or the microsomal fractions from cells reported that changes in the lipid environment greatly influence γ-secretase functions [24,25,26,27,28,29], including longer vs. shorter Aβ production by γ-secretase [27,28]. This cell-free in vitro platform allows precise control of various lipid components such as fatty acyl chain length, saturation, and polar headgroup types and therefore determines the effect of these variables on γ-secretase functioning. These studies uncovered that short fatty acyl chains and thus thinner membrane, cis-fatty acyl chains, and several headgroups are closely associated with decreased γ-secretase processivity and predominant longer Aβ production [27,28]. Furthermore, a previous study treated CHO cells with phospholipids containing different lengths or saturation of fatty acyl chains, and it showed that γ-secretase processivity is decreased in the intact shorter fatty acyl chains-treated cells [27]. If γ-secretase cleaves APP C99 in the endo-lysosomal compartments, endosome and lysosome membrane properties are expected to correlate well with γ-secretase function in live/intact cells.

In the present study, we employed live-cell fluorescence imaging and complementary biochemical subcellular fractionation assays to determine the characteristics of endosome and lysosome membranes. We then compared the endosome and lysosome membrane properties and γ-secretase processivity between primary neurons and CHO cells. Surprisingly we found that the endosome and lysosome membrane in primary neurons is highly disordered compared to that in CHO cells. Moreover the endosome and lysosome membrane in primary neurons is more permeable than in CHO cells. Importantly, we found the predominant production of longer (e.g., Aβ42) compared to shorter Aβ (e.g., Aβ38) in primary neurons, whereas CHO cells exhibit the opposite result. Our results provide further evidence that γ-secretase cleaves APP C99 in the endosomes and lysosomes and add new information that γ-secretase function is tightly associated with the lipid membrane properties of the endo-lysosomal compartments in live/intact neurons. In that sense, increased longer Aβ production uncovered in the non-raft-associated fraction and tightly related to the Braak staging in the AD brain [30] could be due to the altered membrane lipid properties of the neurons.

## 2. Materials and Methods

### 2.1. Antibodies and Reagents

The anti-cathepsin B antibody was from Abcam (Cambridge, UK), the anti-Na^+^/K^+^-ATPase antibody was from MilliporeSigma (Burlington, MA, USA), and the anti-GAPDH and β-tubulin antibodies were from Cell Signaling Technology, Inc (Dover, MA, USA). LipidORDER^TM^ [31] was purchased from DiagnoCine (Hackensack, NJ, USA). LysoPrime Green^TM^ was from Dojindo Molecular Technologies, Inc (Rockville, MD, USA). 4-hydroxy-2-nonenal (HNE) and L-leucyl-L-leucine methyl ester (LLOMe) were purchased from MilliporeSigma. The AAV packaging the C99 YPet-TurquoiseGL (C99 Y-T) [20] was developed using University of Pennsylvania Gene Therapy Program vector core (Philadelphia, PA, USA; 4.95 × 10^13^ GC/mL).

### 2.2. Cell Culture

Primary neurons were obtained from CD1 mouse embryos. The neurons were dissociated using the Papain Dissociation System (Worthington Biochemical Corporation, Lakewood, NJ, USA) and maintained for 12–14 days in vitro in Neurobasal medium with a 2% B27 supplement, 1% GlutaMAX Supplement, and 1% Penicillin Streptomycin (Thermo Fisher Scientific, Waltham, MA, USA). The neuron prep procedure was approved by the Animal Care and Use Committee in MGH (2006N000026).

CHO cells, obtained from ATCC (Manassas, VA, USA), were cultured in Opti-MEM Reduced Serum Medium (Thermo Fisher Scientific, Waltham, MA, USA) supplemented with 5% FBS (Atlanta Biologicals Inc, Flowery Branch, GA, USA). The cells were monitored for mycoplasma contamination every 2 months and authenticated using STR profiling.

### 2.3. Plasmid DNA and Transfection

pLAMP1-emiRFP670 (LAMP1-670) was obtained from Addgene (plasmid #136570) [32]. Lipofectamine 3000 (Thermo Fisher Scientific, Waltham, MA, USA) was used to transfect plasmid into CHO cells.

### 2.4. Subcellular Fractionation and Western Blotting

The cytoplasmic extraction buffer (CEB) containing protease and a phosphatase inhibitor cocktail in the Subcellular Protein Fractionation Kit for Cultured Cells (Thermo Fisher Scientific, Waltham, MA, USA) was added to the pellet of primary neurons or CHO cells, followed by gentle pipetting 5 times to lyse the cells. The lysed cells were incubated at 4 °C for 10 min with gentle rotation. Then the cell lysates were centrifuged at 500× *g* for 5 min, and the supernatants were collected (Fraction #1). The membrane extraction buffer (MEB) was added to the CEB-insoluble pellets, and the tubes were vortexed for 5 s on the highest setting. Then the lysed pellets were further incubated at 4 °C for 10 min with gentle rotation. After centrifuging at 3000× *g* for 5 min, the supernatants were collected (Fraction #2). The successful enrichment of Fraction #1 and #2 was verified by the detection of β-tubulin and Na^+^/K^+^-ATPase, respectively. To obtain the total fraction, a RIPA buffer (Sigma-Aldrich, St. Louis, MO, USA) containing protease and a phosphatase inhibitor cocktail was used to lyse the cells and incubated for 30 min. After centrifuging at 14,000× *g* for 20 min, the supernatant was used as the total fraction.

The Pierce BCA Protein Assay Kit (Thermo Fisher Scientific, Waltham, MA, USA) was used to measure protein concentrations. NuPAGE^TM^ LDS Sample Buffer and NuPAGE^TM^ Sample Reducing Agent (Thermo Fisher Scientific, Waltham, MA, USA) were added to the concentration-normalized samples, followed by boiling for 3 min. Then the samples were subjected to SDS-PAGE on NuPAGE^TM^ 4–12% Bis-Tris Protein gels (Thermo Fisher Scientific, Waltham, MA, USA), followed by blotting to nitrocellulose membranes (Thermo Fisher Scientific, Waltham, MA, USA) using the BioRad Wet electroblotting system (BioRad, Hercules, CA, USA). The membranes were incubated with primary and secondary antibodies and developed using the LI-COR Odyssey CLx scanner (LI-COR Biosciences, Lincoln, NE, USA).

### 2.5. Confocal Microscopy

An Olympus FV3000RS Confocal Laser Scanning Microscope (Tokyo, Japan) was used to measure the lipid membrane order and the endo-lysosomal membrane permeability in live cells. The scope was equipped with a heating/CO2 unit (Tokai-Hit, Fujinomiya, Japan) and the TruFocus Z drift compensation module. A 40×/0.95NA objective was used for imaging. A laser at 405 nm was used to excite the LipiORDER^TM^ solvatochromic dye, and the emitted fluorescence was simultaneously detected within 500–540 nm and 550–650 nm. Pseudo-colored images corresponding to the LipiORDER^TM^ 550–650 nm over 500–540 nm ratios were generated in MATLAB (The MathWorks, Natick, MA, USA). For the excitation of LysoPrime Green^TM^, a 488 nm laser was used, and emission was detected within 500–540 nm.

### 2.6. Aβ ELISA

The culturing medium of primary neurons or CHO cells expressing C99 Y-T were replaced with a serum-free medium and incubated for 24 h. Then Aβ42 and Aβ38 levels in the conditioned medium or the corresponding cell lysates were measured using Human Amyloid β (1–42) and Human Amyloid β (1–38) Assay Kits (Immuno-Biological Laboratories, Inc. Fujioka, Gunma, Japan).

### 2.7. Statistical Analysis

Statistical analysis was performed using GraphPad Prism 9 (GraphPad Software, San Diego, CA, USA). The D’Agostino and Pearson omnibus normality test was used to examine the data distribution and the variance equality. Then an unpaired *t*-test or two-way ANOVA was used to compare the data. We repeated at least three independent experiments to ensure the data reproducibility.

## 3. Results

### 3.1. Lipid Membrane Is More Disordered in Primary Neurons Than in CHO Cells

**Figure 1:**
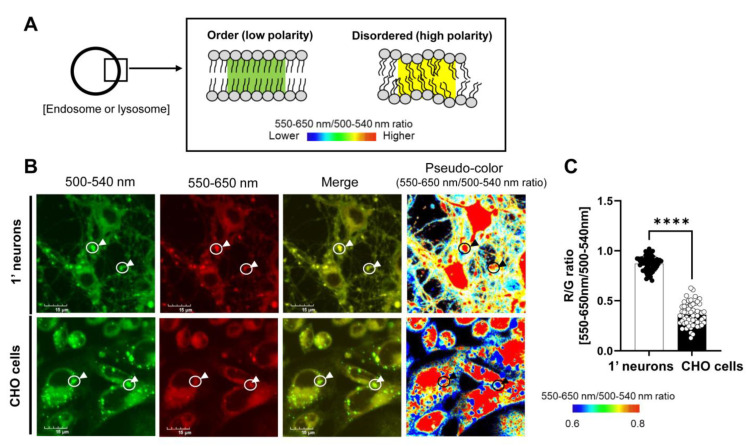
The membrane is more disordered in primary neurons than in CHO cells: (**A**) Schematic presentation of the ratiometric membrane order imaging using LipiORDER^TM^, a highly hydrophobic polarity-sensitive solvatochromic dye, in live cells. LipiORDER^TM^ changes its fluorescence from Green on ordered to Red on disordered lipid membrane (i.e., higher R/G ratios in disordered membrane). (**B**) Primary neurons and CHO cells stained with LipiORDER^TM^ were excited by a 405 nm laser, and the emitted fluorescence within 500–540 nm (G: green) and 550–650 nm (R: red) was simultaneously detected. Confocal images and pseudo-color images corresponding to the R/G ratios are displayed (e.g., high polarity/disordered area: Yellow in confocal and Red in pseudo-color images). Scale bar 15 μm. (**C**) Quantitative analysis of the R/G ratio: region of interest (ROI) was created on the LipiORDER^TM^ positive cytoplasmic vesicles. *n* = 60–66 ROIs from 14–16 cells. Unpaired *t*-test; **** *p* < 0.0001.

Numerous studies, including ours, have suggested that γ-secretase processes APP C99 in the endo-lysosomal compartments, particularly late endosomes and lysosomes [22,23,33,34,35,36,37]. To further ensure that γ-secretase acts in late endosomes and lysosomes and examine whether γ-secretase is associated with the membrane lipid properties of the acidic compartments in live cells, we sought cell types that exhibit different lipid membrane characteristics. We first compared the membrane polarity between primary neurons and CHO cells. As such, the primary neurons and CHO cells were labeled by a highly hydrophobic, polarity-sensitive solvatochromic dye (LipiORDER^TM^) [31], which shifts its fluorescence from green to red under a high polarity/disordered membrane environment. Polar water molecules were excluded from the ordered membrane lipid bilayer, resulting in a change in local polarity within the membrane. We verified that LipiORDER^TM^ stains the plasma membrane and the membrane of cytoplasmic vesicles (arrowhead) in live cells (Figure 1B). Moreover, the solvatochromic dye was in part co-localized with LAMP1-miRFP670 (LAMP1-670), a lysosome marker [32] (Supplementary Figure S1A). Furthermore we found that L-leucyl-L-leucine methyl ester (LLOMe: a lysosomotropic agent) treatment significantly increases the size of LipiORDER^TM^ positive puncta (Supplementary Figure S1B). Then we excited the labeled cells with a 405 nm laser and simultaneously detected the emitted fluorescence within 500–540 nm (G: green) and 550–650 nm (R: red) using a confocal microscope. We drew regions of interest (ROIs) on the LipiORDER^TM^ positive vesicular compartments within the cells, and the ratio of 550–650 nm emission over that of 500–540 nm (i.e., R/G ratio) was calculated to quantitatively assess polarity within the membrane, with an increased R/G ratio indicating higher polarity and thus a more disordered lipid membrane (Figure 1A,B). Interestingly, we found significantly higher R/G ratios in primary neurons than CHO cells (Figure 1B,C). These results suggest that the lipid membrane of cytoplasmic compartments, that presumably include endosomes and lysosomes, is more disordered in primary neurons than in CHO cells.

### 3.2. Endosome and Lysosome Membrane in Primary Neurons Is More Permeable Than that in CHO Cells

**Figure 2:**
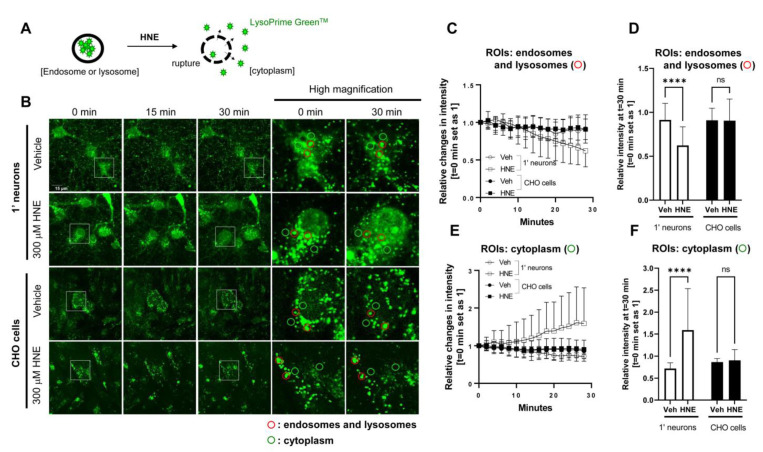
Time-lapse live-cell imaging suggests a more permeable endosome and lysosome membrane in primary neurons than in CHO cells: (**A**) Schematic presentation of HNE-induced endosome and lysosome membrane rupture, resulting in the leakage of LysoPrime Green^TM^ into the cytoplasm. (**B**) Primary neurons and CHO cells were pre-incubated with LysoPrime Green^TM^ to label endosomes and lysosomes. Then the cells were treated with vehicle control or 300 μM HNE to induce the endo-lysosomal membrane rupture, followed by monitoring LysoPrime Green^TM^ fluorescence in endosomes and lysosomes (ROIs: red) and cytoplasm (ROIs: green) for 30 min. Scale bar 15 μm. The relative changes in LysoPrime Green^TM^ fluorescence (time point 0 sets as 1) were quantified in endosomes and lysosomes (**C**) and in the cytoplasm (**E**). After a total of 30 min post-HNE treatment, primary neurons exhibited significantly decreased LysoPrime Green^TM^ fluorescence in endosomes and lysosomes (**D**) and increased fluorescence in the cytoplasm (**F**). On the other hand, CHO cells did not show such changes (**D**,**F**). *n* = 20 ROIs. Two-way ANOVA. **** *p* < 0.0001.

Next we developed a live-cell imaging assay to quantitatively assess endosome and lysosome membrane permeability. Primary neurons and CHO cells were incubated with LysoPrime Green^TM^, a pH-resistant, endosome and lysosome-labeling fluorescent dye, followed by treatment with the 4-hydroxy-2-nonenal (HNE, 300 μM) to induce the rupture of the endosome and lysosomal membrane [38]. We previously showed that HNE treatment alters the structure of γ-secretase and increases the ratio of Aβ42 over Aβ40 in neurons [39]. We then monitored changes in the LysoPrime Green^TM^ fluorescence intensity in endosomes, lysosomes, and in the cytoplasm before and during 30 min of HNE or vehicle treatment. Lastly we compared the changes in the fluorescence intensity between primary neurons and CHO cells (Figure 2A,B). We found that vehicle treatment did not alter fluorescence in endo/lysosomes and cytoplasm of both cell types (Figure 2B,C,E). On the other hand, HNE treatment decreased the LysoPrime Green^TM^ fluorescence in endosomes and lysosomes and increased its fluorescence in the cytoplasm in primary neurons; however such changes were not detected in CHO cells (Figure 2B,C,E). At the 30 min time point, primary neurons treated with HNE exhibited significantly lower relative fluorescent intensity (*t* = 0 set as 1) in endosomes and lysosomes (Figure 2D) and higher relative intensity in the cytoplasm (Figure 2F) compared to vehicle-treated neurons. In contrast, CHO cells did not change the LysoPrime Green^TM^ fluorescence intensity in endosomes, lysosomes, and the cytoplasm after HNE treatment (Figure 2D,F). Of note, we confirmed that 300 μM HNE required 45–50 min to cause endosome and lysosome membrane permeabilization in CHO cells (Supplementary Figure S2).

**Figure 3:**
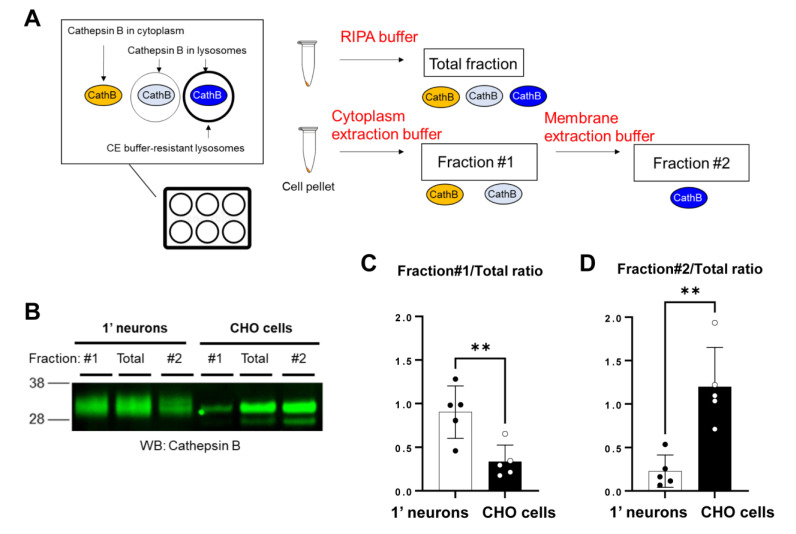
Cell fractionation reveals that lysosomal membrane is more permeable in primary neurons than in CHO cells: (**A**) Schematic presentation of the biochemical assay to measure lysosomal membrane integrity. In this analysis, primary neuron or CHO cell pellets are first solubilized in the cytoplasmic extraction buffer (CEB), followed by centrifugation. The supernatant contains (1) Cathepsin B localized in the cytoplasm of the cells and (2) Cathepsin B localized within the naïve lysosomes in the cells but solubilized by the CEB (Fraction #1). On the other hand, the CEB insoluble pellets lysed by the membrane extraction buffer (MEB) contain (3) Cathepsin B localized within the intact CEB-resistant lysosomes (Fraction #2). RIPA buffer was used to extract all Cathepsin B (total fraction). (**B**) A representative Western Blot image indicated the different lysosomal membrane permeability between primary neurons and CHO cells. The ratio of Cathepsin B levels in Fraction #1 over the total fraction was significantly higher (**C**), while the Fraction #2/total ratio was substantially lower (**D**) in primary neurons compared to those in CHO cells. *n* = 5 independent experiments; unpaired *t*-test; ** *p* < 0.01.

To further validate the findings from live-cell imaging, we performed subcellular fractionation, followed by Western Blotting to measure the level of Cathepsin B: a lysosomal hydrolase (Figure 3A). In this biochemical analysis, primary neurons and CHO cells were solubilized in the cytoplasmic extraction buffer (CEB) from a commercially available subcellular fractionation kit. As a result, we detected (1) the Cathepsin B which was present in the cytoplasm of the cells and (2) the Cathepsin B which was localized within the naïve lysosomes of the cells that the CEB permeabilizes during solubilization (Fraction #1 in Figure 3A). Then CEB insoluble pellets were lysed using the membrane extraction buffer (MEB), allowing us to detect (3) the Cathepsin B which was localized within the intact lysosomes that are resistant to the CEB (Fraction #2). Therefore, the relative Cathepsin B levels in Fraction #1 over the RIPA buffer-extracted total fraction and those in Fraction #2 over the RIPA total fraction can be used as indicators of the lysosomal membrane integrity. Successful enrichment of Fraction #1 and #2 was verified by detecting β-tubulin and Na^+^/K^+^-ATPase, respectively (Supplementary Figure S3). We found that the ratio of Cathepsin B levels in Fraction #1 over the total fraction was significantly higher in primary neurons than CHO cells (Figure 3B,C). Moreover the Fraction #2/total ratio was lower in primary neurons than in CHO cells (Figure 3B,D). This trend was similarly detected in the cells treated with HNE (Supplementary Figure S4). Collectively these results suggest that primary neurons exhibit increased endosome and lysosome membrane permeability compared to CHO cells.

### 3.3. Predominant Production of Longer Aβ in Primary Neurons Compared to CHO Cells

**Figure 4:**
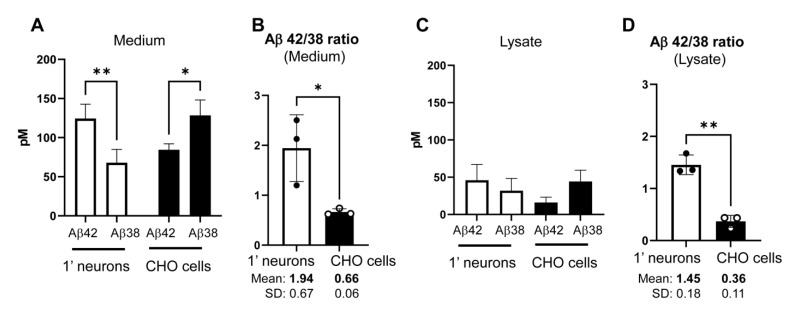
Higher Aβ42/38 ratio in the conditioned medium and lysate of primary neurons than those in CHO cells: Aβ42 and 38 levels in the conditioned medium (**A**) and lysate (**C**) of primary neurons or CHO cells expressing the C99 Y-T were measured using human Aβ ELISA. *n* = 3 independent experiments; Two-way ANOVA; * *p* < 0.05. (**B**,**D**) Aβ42/38 ratio was calculated and compared between the two cell types. Unpaired *t*-test; * *p* < 0.05; ** *p* < 0.01.

Previous studies using proteoliposomes showed that the generation of longer vs. shorter Aβ by γ-secretase is tightly associated with lipid membrane properties [27,28]. Our new results suggest that the endosome and lysosome membrane in primary neurons is more disordered (Figure 1) and permeable (Figure 2 and Figure 3) than in CHO cells. We lastly examined if primary neurons and CHO cells exhibit different γ-secretase processivity and thus longer vs. shorter Aβ generation. As such, we expressed C99 YPet-TurquoiseGL (C99 Y-T) [20], which encoded human APP C99 in primary neurons and CHO cells and performed ELISA to measure human Aβ42 and Aβ38 levels in the conditioned medium and lysate of the cells. Interestingly, we uncovered that primary neurons produce and secrete more Aβ42 than Aβ38 (Figure 4A), resulting in significantly higher Aβ42/38 ratios in the medium (average Mean ± SD: 1.94 ± 0.67) (Figure 4B). In contrast, CHO cells favored Aβ38 over the Aβ42 production and thus displayed significantly lower Aβ42/38 ratios (Mean ± SD: 0.66 ± 0.06) compared to primary neurons (Figure 4A,B). Furthermore, we detected significantly lower Aβ42/38 ratios in the lysate of CHO cells (Mean ± SD: 0.36 ± 0.11) than in primary neurons (Mean ± SD: 1.45 ± 0.18) (Figure 4C,D). Of note, a recent study has performed an in vitro aggregation kinetics analysis of Aβ42 and Aβ38 peptides mixtures and showed that Aβ38 significantly inhibits Aβ42 aggregation below the range of the Aβ42/38 ratio = 0.6 [40]. Therefore we expect that Aβ38 inhibits Aβ42 aggregation in CHO cells but not in primary neurons. Altogether these results suggest that endosome and lysosome membrane properties are tightly correlated with γ-secretase functioning. Furthermore they support the idea that γ-secretase cleaves APP C99 in the endo-lysosomal compartments in live/intact cells.

## 4. Discussion

Our recent development of genetically encoded FRET-based biosensors has enabled us to detect γ-secretase activity in live cells with subcellular resolution [20,21]. By expressing the biosensors in neurons, we showed that γ-secretase processes APP C99 primarily within the late endosome and lysosome membrane, resulting in the generation and enrichment of Alzheimer’s-linked β-amyloid (Aβ) peptides in the same subcellular compartments [22,23]. γ-Secretase is embedded within the membrane lipid bilayer, and previous studies employing cell-free lipid vesicles reported that γ-secretase processivity and thus the generation of longer vs. shorter Aβ is tightly associated with lipid membrane properties [27,28]. Therefore we hypothesized that γ-secretase-mediated longer vs. shorter Aβ generation correlates with endosome and lysosome membrane properties in live/intact cells. In the present study, we employed live-cell fluorescence imaging and complementary biochemical analysis and uncovered that endosome and lysosome membranes in primary neurons are more disordered (Figure 1) and permeable (Figure 2 and Figure 3) than those in CHO cells. Furthermore, we found the predominant production of aggregation-prone Aβ42 as opposed to Aβ38 in primary neurons, whereas CHO cells exhibited the opposite result (Figure 4). Such a functional correlation between endosome and lysosome membrane properties and γ-secretase functioning further supports the conclusion that γ-secretase cleaves APP C99 in the endo-lysosomal compartments in cells.

Aβ accumulation, so-called senile plaques, is a diagnostic marker of AD [41,42,43]. The appearance of senile plaques is the earliest pathological alteration in the AD brain, preceding other changes such as synaptic dysfunction, neuroinflammation, neurofibrillary tangles, and neuronal loss [43]. Therefore the removal of Aβ by antibodies or blocking Aβ production by β- and γ-secretase inhibitors has been tested in the clinical trials of AD. Recent clinical trials utilizing antibodies against Aβ show promising results, suggesting that Aβ removal could slow cognitive worsening [14,15]. Nevertheless little is known about why Aβ accumulates in the brain and what molecular and cellular event(s) cause Aβ deposition in sporadic cases. Impaired Aβ clearance rather than its production is reported to dominate in the sporadic AD brain and may contribute to amyloid plaque accumulation [44]. However, a recent study analyzing sporadic cases with different Braak senile plaque stages reported that shorter and longer Aβ production decreases and increases with the advancing of the Braak stages, respectively [30]. Given that longer Aβ (e.g., Aβ42, Aβ43) are highly prone to aggregation [45,46,47], the extent of long Aβ production may be one of the factors instigating Aβ plaques deposition in sporadic AD brains.

γ-Secretase needs to process APP C99 three or four times to generate longer (e.g., Aβ42) or shorter Aβ peptides (e.g., Aβ38), respectively [6,7]. However why some endogenous γ-secretase complexes trim APP C99 only three times and generate longer Aβ while the others cleave four times and produce shorter Aβ remains unclear. Our findings suggest that primary neurons, which favor longer Aβ generation (Figure 4), reveal a more disordered endo-lysosomal membrane (Figure 1) than CHO cells that predominantly produce shorter Aβ. A recent systematic study demonstrated that the membrane permeability of small molecules increases in the thinner membrane, which is the membrane with a more significant degree of fatty acyl chains unsaturation and decreased cholesterol content [48]. In addition, by employing proteoliposomes, other studies highlighted that the γ-secretase within the thinner membrane and/or the membrane with unsaturated fatty acyl chains favors long Aβ production [27,28]. Our findings are in line with these reports, demonstrating that primary neurons exhibit a higher degree of membrane disorder (Figure 1), increased endosome and lysosome membrane permeability (Figure 2 and Figure 3), and decreased γ-secretase processivity, and therefore favor longer over shorter Aβ generation compared to CHO cells (Figure 4). Altogether, it appears that γ-secretase may predominantly produce longer Aβ within the disordered region of the membrane in intact cells, likely because of a thinner bilayer and/or loss of lipid packing due to, for instance, lower cholesterol levels. Nevertheless, Aph1, one of the components of γ-secretase, has three isoforms in murine and, of note, it is well established that Aph1a and Aph1b differently impact the generation of longer and shorter Aβ [49,50,51,52]. Whether primary neurons and CHO cells express different levels of γ-secretase complexes containing Aph1a or Aph1b and whether this difference causes the altered Aβ generation in the two cell types should be investigated in future studies.

Numerous mutations causing early-onset AD have been discovered in the genes encoding APP and Presenilin (PSEN) (https://www.alzforum.org/mutations, accessed on 14 January 2023). PSEN is the catalytic component of γ-secretase responsible for the cleavage of APP and generation of Aβ within the lipid membrane bilayer [4,5]. APOE is a major genetic risk factor for late-onset AD, which is responsible for distributing cholesterol and other lipids to the lipid membrane bilayer of neurons [53]. Neurofibrillary tangles, a key pathological hallmark and used as an AD diagnostic marker [41,42,43], are known to be associated with the cell-to-cell propagation of Tau seed via a prion-like manner [reviewed in [54]]. Tau proteins must cross the lipid membrane bilayer in the propagating process, most likely within the endo-lysosomal compartments [55,56]. Based on these facts, it would be intriguing to speculate that alteration in the lipid membrane plays a pivotal role in the development of AD pathology. Importantly, accumulating evidence demonstrates that the spreading of Tau pathology is accelerated in the presence of amyloid pathology [57,58,59]. Considering that aggregation-prone longer Aβ generation is tightly associated with the appearance of Aβ plaques, the presence of senile plaques can be an indicator of altered membrane properties of the neurons (i.e., disordered, thinner, and/or less packed membrane). Then Tau protein may preferentially propagate into the neurons bearing a disordered/thinner lipid membrane bilayer. Although extensive evidence would be required to prove the following hypothesis, it is plausible that the pathological alterations detected in the AD brain (i.e., plaques and tangles) could be the consequences of the altered lipid membrane bilayer.

## 5. Conclusions

In conclusion, our new study employing new live-cell imaging and biochemical assays highlights the functional correlation between lipid membrane properties and γ-secretase processivity in live/intact cells. Furthermore it adds new evidence that γ-secretase cleaves APP C99 in the endo-lysosomal compartments of live/intact neurons and provides new insight into the regulation of γ-secretase and its consequences. Investigation focusing on the lipid membrane bilayer in the AD brain may open a new avenue to develop a novel therapeutic approach and refine the diagnostic approach to AD.

## Data Availability

All data are presented in Figure 1, Figure 2 and Figure 3 and Supplementary Figures S1–S3.

## Supplementary Materials

The following supporting information can be downloaded at: https://www.mdpi.com/article/10.3390/s23052651/s1, Figure S1: Lysosomes are labeled by LipiORDER^TM^; Figure S2: HNE-induced rupture of endosomes and lysosomes in CHO cells; Figure S3: Cell fractionation using the cytoplasmic (CEB) and membrane extraction buffer (MEB). Figure S4: Cell fractionation post-HNE treatment.
