# Supplementary material for: Endosome and Lysosome Membrane Properties Functionally Link to γ-Secretase in Live/Intact Cells
Source: Sensors (Basel). 2023 Feb 28;23(5):2651. doi: 10.3390/s23052651 (PMC10007619; doi:10.3390/s23052651)
Supplement: Supplementary file 1 [file sensors-23-02651-s001.zip › sensors-2193057-supplementary.pdf]

Supplemental Figure S1

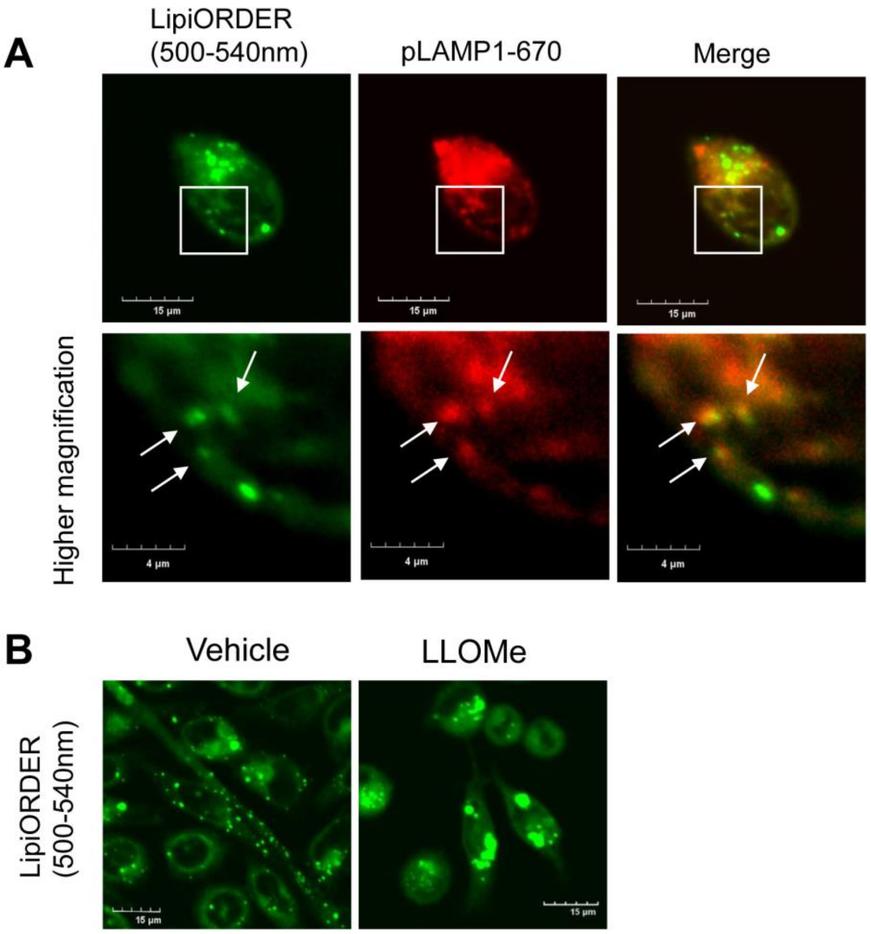

Lysosomes are labeled by LipiORDER™: (**A**) CHO cells were transfected with pLAMP1 670, a lysosome marker, followed by staining with LipiORDER™, a polarity sensitive solvatochromic dye. Then, the cells were excited by 405 nm and 640 nm lasers, and the emitted fluorescence within the 500-540 nm (LipiORDER™) and 660-690 nm (LAMP1 670) was detected. Scale bar 15 μm. Lower panels show high magnification images corresponding to the squares in the upper panels. Scale bar 4 μm. (**B**) CHO cells were treated with either 100 μM L-Leucyl-L-Leucine methyl ester (LLOMe) or vehicle control for 30 minutes, followed by staining with LipiORDER™. LLOMe treatment significantly increased the size of LipiORDER™ positive puncta. Scale bar 15 μm.

Supplemental Figure S2

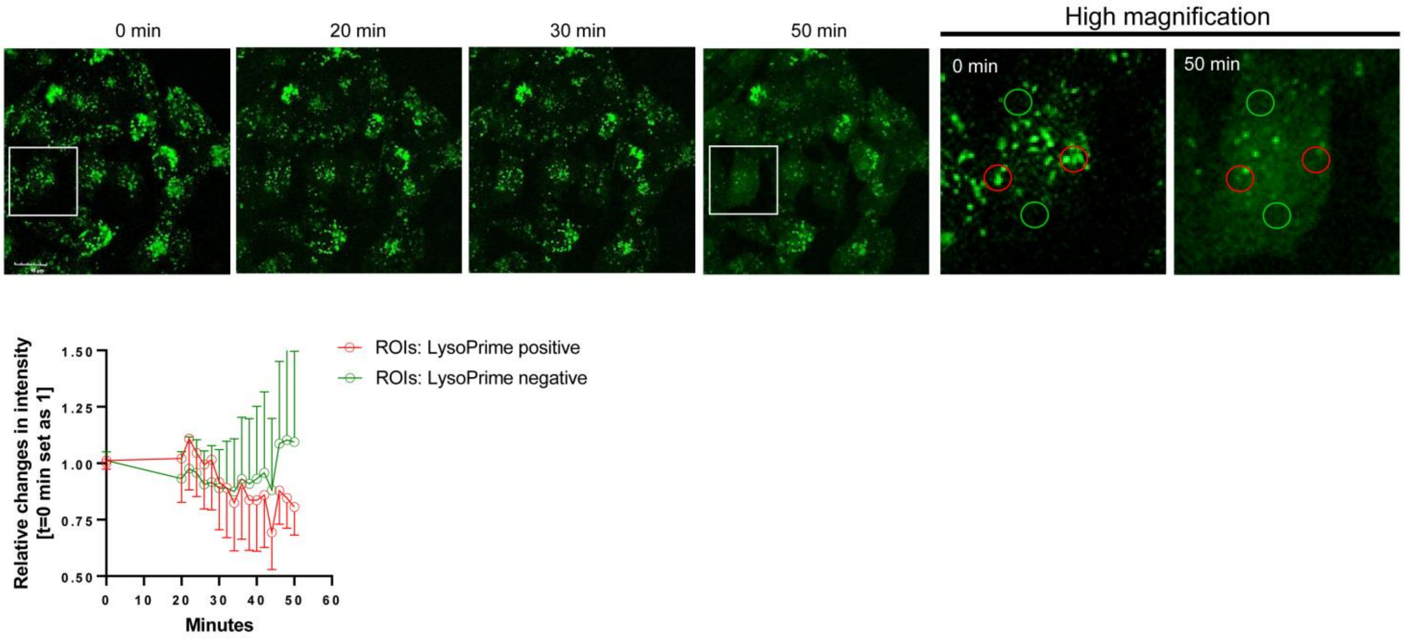

HNE-induced rupture of endosomes and lysosomes in CHO cells: CHO cells were pre-incubated with LysoPrime Green^{TM} to label endosomes and lysosomes. Then, the cells were treated with 300 μM HNE to induce the endo-lysosomal membrane permeabilization, followed by monitoring LysoPrime^{TM} fluorescence for 50 min. Scale bar 15 μm. ROIs were created on the LysoPrime^{TM} positive endo-lysosomal compartments (e.g., red circles in high magnification images) and negative cytoplasmic (e.g., green circles) areas, and the relative changes in fluorescence within 500-540 nm range (time point 0 sets as 1) were quantified. N=20 ROIs.

Supplemental Figure S3

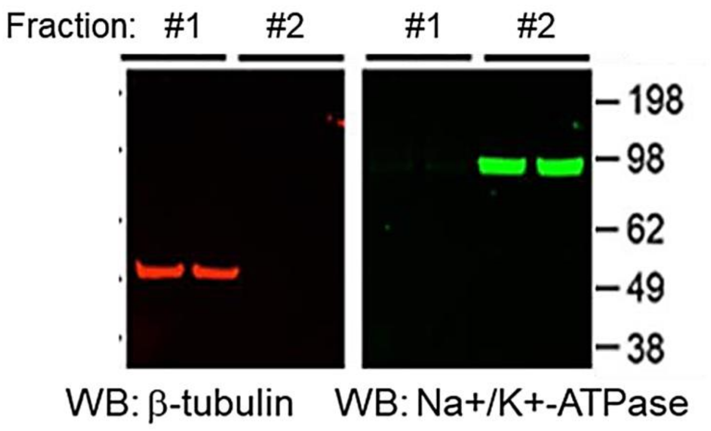

<u>Cell fractionation using the cytoplasmic (CEB) and membrane extraction buffer (MEB)</u>: The detection of β-tubulin (a cytoplasmic marker) and Na^+/K^+-ATPase (a membrane marker) verifies the successful extraction of Fractions#1 and #2 in primary neurons and CHO cells.

Supplemental Figure S4

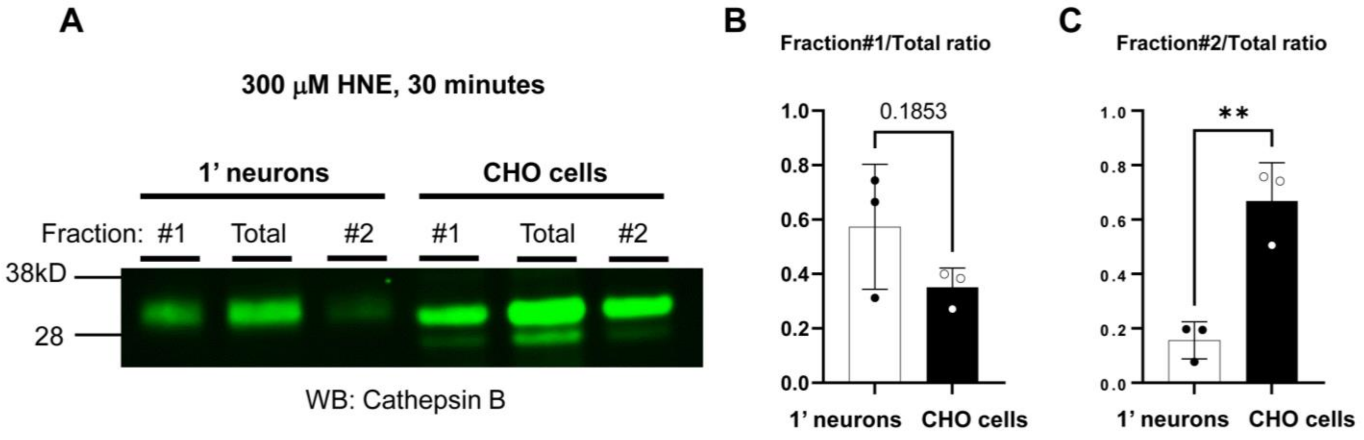

Cell fractionation post-HNE treatment: (**A**) Primary neurons and CHO cells were treated with 300 μM HNE for 30 minutes, followed by subcellular fractionation. A representative Western Blot image and band quantification (**B** and **C**) indicated increased lysosomal membrane permeability in primary neurons compared to CHO cells. N=3 independent experiments, unpaired t-test, **p<0.01.
